# Expression of olfactory receptor genes in non-olfactory tissues in the developing and adult zebrafish

**DOI:** 10.1038/s41598-023-30895-3

**Published:** 2023-03-21

**Authors:** Dania Jundi, Jean-Pierre Coutanceau, Erika Bullier, Soumaiya Imarraine, Ziad Fajloun, Elim Hong

**Affiliations:** 1 INSERM, CNRS, Neurosciences Paris Seine-Institut de Biologie Paris Seine (NPS-IBPS), Sorbonne Université, 75005 Paris, France; 2 Laboratory of Applied Biotechnology (LBA3B), Azm Center for Research in Biotechnology and Its Applications, EDST, Lebanese University, Tripoli, 1300 Lebanon; 3 CNRS, Laboratoire Jean Perrin-Institut de Biologie Paris Seine (LJP-IBPS), Sorbonne Université, 75005 Paris, France; 4 Department of Biology, Faculty of Sciences 3, Campus Michel Slayman, Lebanese University, Tripoli, 1352 Lebanon

**Keywords:** Developmental biology, Neuroscience, Cellular neuroscience

## Abstract

Since the discovery of olfactory receptor (OR) genes, their expression in non-olfactory tissues have been reported in rodents and humans. For example, mouse OR23 (mOR23) is expressed in sperm and muscle cells and has been proposed to play a role in chemotaxis and muscle migration, respectively. In addition, mouse mesencephalic dopaminergic neurons express various ORs, which respond to corresponding ligands. As the OR genes comprise the largest multigene family of G protein-coupled receptors in vertebrates (over 400 genes in human and 1000 in rodents), it has been difficult to categorize the extent of their diverse expression in non-olfactory tissues making it challenging to ascertain their function. The zebrafish genome contains significantly fewer OR genes at around 140 genes, and their expression pattern can be easily analyzed by carrying out whole mount in situ hybridization (ISH) assay in larvae. In this study, we found that 31 out of 36 OR genes, including *or104-2, or108-1, or111-1, or125-4, or128-1, or128-5, 133-4, or133-7, or137-3* are expressed in various tissues, including the trunk, pharynx, pancreas and brain in the larvae. In addition, some OR genes are expressed in distinct brain regions such as the hypothalamus and the habenula in a dynamic temporal pattern between larvae, juvenile and adult zebrafish. We further confirmed that OR genes are expressed in non-olfactory tissues by RT-PCR in larvae and adults. These results indicate tight regulation of OR gene expression in the brain in a spatial and temporal manner and that the expression of OR genes in non-olfactory tissues are conserved in vertebrates. This study provides a framework to start investigating the function of ORs in the zebrafish brain.

## Introduction

The olfactory system is essential for the survival of the individual and species, as it plays a crucial role in detecting food, warning signals and in reproduction. In humans, the nasal cavity contains the olfactory epithelium, which consists of olfactory sensory neurons (OSN). The dendrites of the OSNs contain cilia, which are densely populated by olfactory receptors (ORs), that protrude into the mucosal layer^1^. The ORs belong to the G protein-coupled receptor (GPCR) superfamily, which consists of the largest class of membrane proteins in the human genome^2,3^. There are around 1000 OR genes in rodents, 400 in human and 140 in zebrafish^4,5^. While ORs are generally thought to be expressed specifically in OSNs, studies have demonstrated their expression in non-chemosensory tissues, including heart, lung, skin, liver, muscle and sperm^6–8^. Growing number of studies have revealed that the ORs expressed in non-olfactory tissues are indeed functional. For example, mouse OR23 (mOR23) plays a role in regulating migration and adhesion in muscle cells in vitro^9^ and chemotaxis in sperm motility^10^. Gene array studies have also shown the expression of various ORs in mouse and human dopaminergic neurons in the substantia nigra^11^. Moreover, Olfr1505 and Olfr287 were found in mesencephalic dopaminergic neurons, which show a calcium response to ligands for Olfr287, S- and R- carvones and decanoic acid^12^. Furthermore, cancer cells upregulate OR expression suggesting odorants could play a role in cancer progression^13^. Indeed, derivatives of androstenone activate OR51E2 receptors causing intracellular calcium increase, resulting in decreased proliferation of prostate cancer cells^14^. Therefore, understanding the spatial and temporal regulation of OR expression could help with health and disease progression. However, due to the large number of OR genes, it is difficult to systematically categorize their expression in non-olfactory tissues in mammals.

The zebrafish (*Danio rerio*) genome contains a smaller number of OR genes at around 140 genes, which are expressed from 24 h post fertilization^15–17^. The small size and transparency of larvae facilitate whole mount in situ hybridization, allowing detailed analyses of transcript expression throughout the whole animal. Here, we mined RNA-seq data from adult zebrafish brain and carried out in situ hybridization analysis of OR transcripts in larvae and adult brains. We found that transcripts for *or103-5, or 104-2, or108-1, or128-5* and *or137-3* were expressed throughout the body, including muscle, pharynx, eyes and the brain in larvae. In addition, we found multiple OR genes, including *or102-3, or103-1, or103-5, or104-1, or105-1, or109-9* and *or112-1*, were expressed in specific regions in the adult brain, such as the optic tectum, hypothalamus and the habenula. We verified these results by performing RT-PCR in larvae without olfactory epithelium and in adult brain extractions. These results demonstrate that ORs are expressed in various tissues throughout the body in larvae and in distinct regions in the adult brain, providing the framework to start dissecting the function of evolutionarily conserved ORs in non-olfactory tissues.

## Materials and methods

### Zebrafish

Wild-type AB strain larvae and adult (around 1 year old) zebrafish and *Tg(OMP*^*6k*^*:GFP)*^*rw033*^ larvae^18^ (referred to as *Tg(OMP:GFP)*) were used in this study. Animals were raised and housed at 28 °C in a 14/10 h light/dark cycle at the fish facility approved by the French “Service for animal protection and health” (A-75-05-25). Experiments were performed in agreement with the European Directive 210/63/EU on the protection of animals used for scientific purposes and the French application decree ‘Décret 2020-118’ and follows the recommendation in the ARRIVE guidelines. All experiments on larvae and adult zebrafish have been approved by the ethical committee “Comité d’éthique Charles Darwin” (APAFIS#27355-2021012213387351 v4). Phenylthiourea (0.2 mM) was added daily to fish water from 24 h post fertilization to prevent pigmentation in larvae.

### RNA in situ hybridization

Plasmids for full-length coding regions of ORs were described in^19,20^. Antisense RNA probes were synthesized using SP6 or T7 polymerases (Promega) and digoxigenin-labeled UTP (Roche). Whole mount colorimetric in situ hybridization (ISH) in zebrafish larvae was performed as described in^21^. Briefly, probes were tagged with uridine-5’-triphospate UTP-digoxigenin and incubated with the larvae overnight at 70 °C in 50% formamide-containing hybridization solution. Hybridized probes were then visualized using alkaline phosphatase-conjugated antibodies that hydrolyze 5-bromo-4-chloro-3-indolyl-phosphate (BCIP) with 4-nitro blue tetrazolium (NBT). Larvae were washed in phosphate-buffered saline (PBS) and conserved at 4 °C until imaged. Three to five months old male and female zebrafish were anesthetized in 0.4% tricaine (ethyl-m-aminobenzoate methanesulfonate), decapitated and dissected in cold Ringer’s solution (0.134 M NaCl, 2.9 mM KCl, 1.2 mM MgCl_2_, 8.3 mM HEPES, 10 mM glucose, 2.8 mM CaCl_2,_ pH ~ 7.5). Dissected brains were fixed in 4% paraformaldehyde overnight at 4 °C, then stored in 100% methanol at − 20 °C. ISH was performed as described for larvae above with the following modifications: brains were rehydrated in PBS followed by treatment with 20 μg/ml proteinase K for 50 min then post-fixed in 4% paraformaldehyde at room temperature for 1 h. Sectioned brain slices were mounted on slides and developed using NBT/BCIP treatment in a dark and humid box. Slides were then washed with PBS and mounted with a cover slip with Mowiol (Sigma-Aldrich)^22^.

### Sectioning and microscopy

Larvae or adult brains processed with ISH were mounted in 4% low-melting agarose and sectioned at 35 µm transverse slices using a vibratome (Leica, Inc) in PBS^22^. Larvae processed by ISH were mounted laterally in 1.2% agarose for imaging. Whole larvae and slides were imaged using 5 ×, 10 × or 20 × Objective W "N-Achroplan" water immersion lens on a Zeiss Apo Examiner Upright Microscope equipped with Zeiss Axiocam 305 color microscope camera.

### RNA extraction and cDNA synthesis

RNA was extracted from ten AB male and female adult zebrafish brains, eighty *Tg(OMP: YFP)*^18^ 6-day-old whole larvae and eighty larvae without their nose. The nose (olfactory epithelium) was removed using fine forceps by gently pinching off the anterior forebrain region in anesthesized (0.4% tricaine) larvae. The lack of yellow fluorescent protein (YFP) to ensure that the nose was fully removed was verified under a fluorescence microscope for each larva. Adult zebrafish were dissected on ice in cold Ringer’s solution. Samples were treated with TRIzol Reagent (Invitrogen), washed with phenol/chloroform and precipitated with isopropanol. Extracted RNA was treated with DNAse I and cDNA synthesized using SuperScript™ III First-Strand Synthesis (Invitrogen) according to manufacturer’s instructions.

### Analysis of olfactory receptors by RT-PCR

The following primers were used for RT-PCR: *or102.3 (fwd—GCCCTATTGTGCTTCTAATGTGG, rev—ATTGGAGGGCCCACAACTAT:* 138 bp*), or104.1 (fwd—TGTTTGTGCTCCTCGTCCA, rev—CGCATTCGGGACGTTAGAAG:* 191 bp*), or104.2 (fwd—TGTCTAATGTTGTGAAGTGTGC, rev- CCGCTGTCAGGCTGTAGTAT:* 167 bp*), or105.1 (fwd—GCTGGAAGAGTTTTGCCACG, rev—CTGATCTCCTGTGTCCGCAA:* 198 bp*), or106.1 (fwd—TTGGTGATTTCACAGAGTGTATGG, rev—TAACTGAGCAGGACAACAAGGT:* 411 bp*), or112.1 (fwd—GGAGCAAACTTCTCGCACAC, rev—TAACTGAGCAGGACAACAAGGT:* 200 bp*), or133.7 (fwd—CTTCGGTCACATGCTTTGGG, rev—AGATGGAAGATGCGCCAAGA:* 556 bp*).* The final PCR products were visualized using 1.2% agarose gel in TAE buffer. gDNA from zebrafish larvae was used as a positive control and sterile water as negative control (results not shown).

## Results

### Olfactory receptor genes are expressed in zebrafish larvae

**Figure 1:**
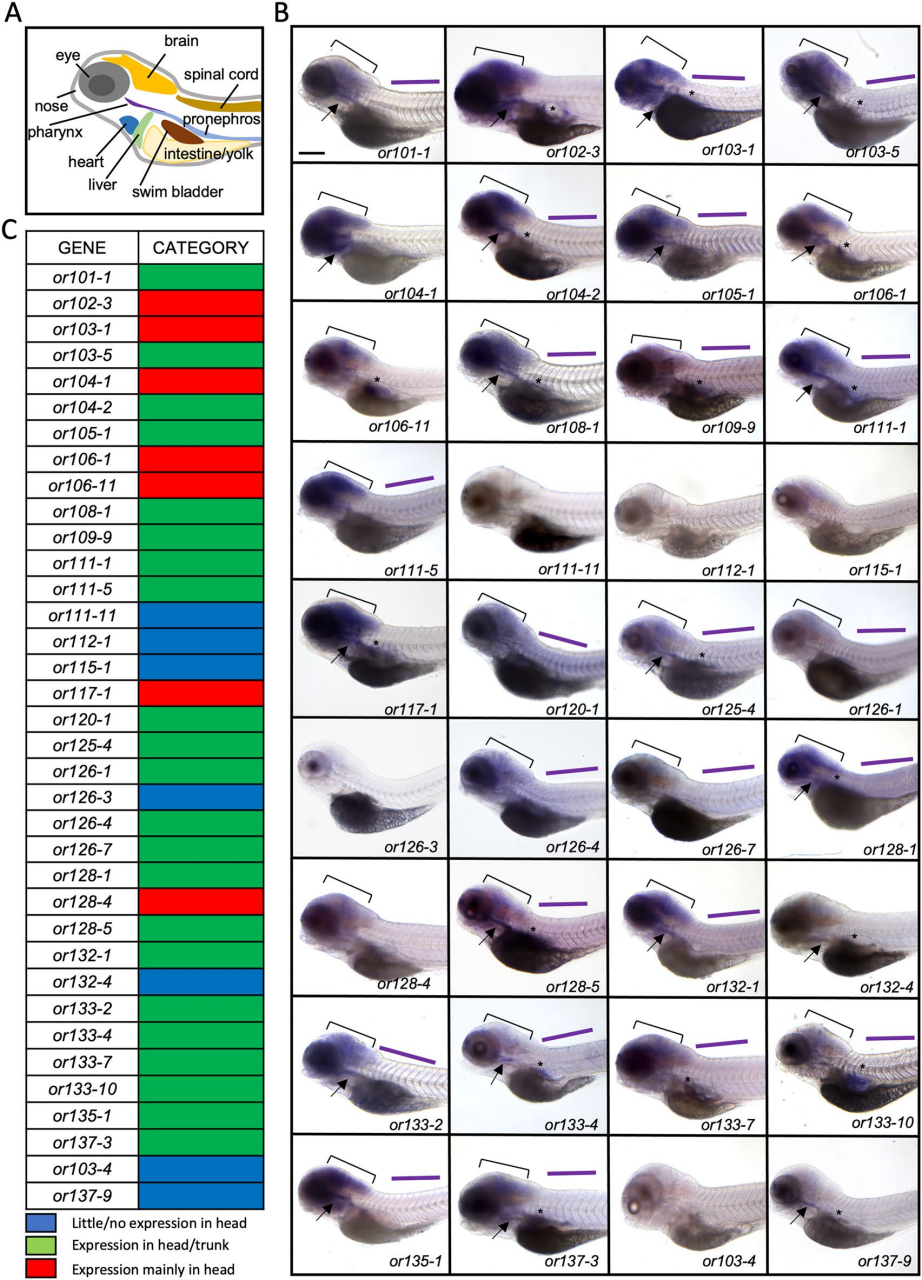
Olfactory receptor genes are expressed throughout the body of zebrafish larvae. (**A**) Schematic of a lateral view of zebrafish larva illustrating different organs. (**B**) Lateral views of 6-day-old larvae processed by whole mount in-situ hybridization showing expression of 36 olfactory receptor genes throughout the head and body. Panels are ordered in numerical order of the gene names, except for *or103-4* and *or137-9,* which were not found in the RNA-seq data and placed at the end. The head (bracket), pharynx (arrow), pancreas (asterisk) and trunk (purple line) are indicated. Scale bar = 200 μm. (**C**) Summary of olfactory receptor gene expression classified into categories (1) little/no expression in the head (blue), (2) expression in the head and trunk (green), (3) strong expression in the head (red).

Compilation of RNA-seq analyses in dissected adult brains from two separate studies (Saraiva et al.^23^, Halpern lab, unpublished) revealed the presence of 97 out of 140 OR transcripts. We randomly chose 44 OR genes (42 ORs present in RNA-seq data + 2 ORs absent from RNA-seq data) and carried out a screen to assay for the expression of these genes in 6-day-old larvae using whole mount ISH. Firstly, to verify the efficacy of the OR probes, we confirmed that 36 OR genes are expressed in the olfactory epithelium of 6-day-old larvae (Fig. S1). 8 OR genes that were not expressed in the olfactory epithelium were excluded from the following study. Next, we analyzed the expression of the 36 OR genes and found that 31 ORs are expressed in non-olfactory tissues throughout the larvae, including the head, trunk, pharynx and pancreas (Fig. 1A,B, Table S1). We carried out a control experiment using anti-sense and sense *or103-1* probes and found significantly lower expression with the sense probe (Fig. S2). The expression profile from the sense probe was used as the standard to classify the OR genes based on their expression profile into 3 categories: (1) absent or low expression throughout the larva (similar to the *or103-1* sense probe: *or111-11, or112-1, or115-1, or126-3, or132-4*), (2) expression in the head and trunk (e.g.* or105-1, or111-5, or133-2, *etc*.*), and (3) expressed mainly in the head (*or102-3, or103-1, or104-1, or106-1, or106-11, or117-1, or128-4*) (see Fig. 1C). The two OR transcripts that were absent in the RNA-seq data (*or103-4* and *or137-9*) displayed little or no expression in the head (Category 1, Fig. 1B,C). The results show that a majority of OR genes found via RNA-seq in adult brain are expressed in non-olfactory tissues in larvae.

### Olfactory receptors are expressed in distinct brain regions in larvae

**Figure 2:**
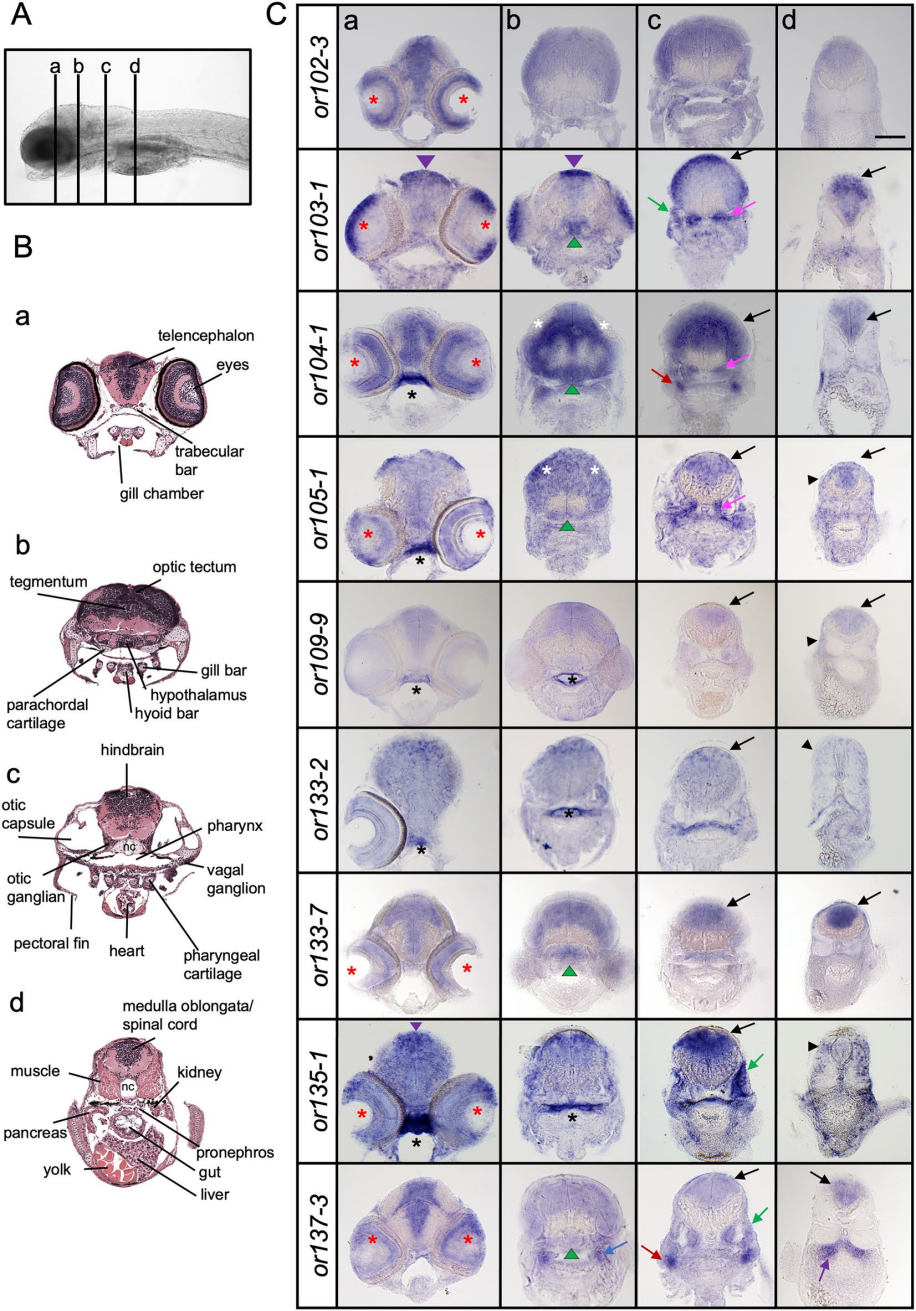
Olfactory receptor genes are expressed in distinct areas in the larval head. (**A**) Lateral view of a zebrafish larva showing the anterior–posterior levels (a–d) of the transverse sections in (**B**) and (**C**). (**B**) Images of transverse sections of 7-day-old larvae with different structures annotated. Sections represent the forebrain (a), midbrain (b), hindbrain (c) and posterior hindbrain (d). Modified from Bio-Atlas (Jake Gittlen Laboratories for Cancer Research). (**C**) Transverse sections of 6–7-day-old zebrafish larvae processed by in situ hybridization showing expression of olfactory receptor genes in different areas of the head. The stainings are localized in the dorsal midline (purple arrowhead), eyes (red asterisk), roof of pharynx (black asterisk), optic tectum (white asterisk), hypothalamus (green arrowhead), otic capsule (green arrow), otic ganglion (pink arrow), anterior lateral line ganglion (blue arrow), pronephros (purple arrow), vagal ganglion (red arrow), hindbrain (black arrow) and muscle (black arrowhead). Scale bar = 100 μm.

To confirm the expression of OR genes in the brain and surrounding areas, we carried out transverse sectioning of larvae expressing categories 2 and 3 ORs. We analyzed the expression of 9 OR genes in the forebrain, midbrain, hindbrain and posterior hindbrain sections (Fig. 2). We found that the staining was not always uniform throughout the sections but localized to distinct regions for some ORs. For example, while the expression of most OR genes tested appeared to be ubiquitous in the forebrain, *or103-1* and *or135-1* transcripts were localized to the more dorsal/peripheral regions. In the midbrain, labeling was observed in the optic tectum (all OR genes) and in the hypothalamus (*or103-1, or104-1, or105-1, or133-7, or137-3*). Staining was also observed in the hindbrain for all tested OR transcripts, albeit *or103-1* and *or135-1* were strongly expressed in the dorsal-part of the hindbrain, while the staining for *or104-1* was stronger in the ventral hindbrain. Most OR gene transcripts were found in the posterior hindbrain except for *or133-2* and *or135-1*. These results show that different ORs are indeed expressed in distinct brain regions in zebrafish larvae.

In the peripheral tissues, labeling was also observed in putative ganglions including the otic ganglion (*or103-1, or104-1, 105-1*), anterior lateral line ganglion (*or137-3*) and/or vagal ganglion (*or104-1*), the roof of the pharynx (*or104-1, or105-1, or109-9, or133-2, or135-1*), otic capsules (*or103-1, or105-1, or135-1, or137-3*), muscle (*or104-1, or105-1, or109-9, or133-2, or135-1*) or the pronephros (*or137-3*). These results indicate that OR genes are also expressed in diverse non-olfactory tissues.

**Figure 3:**
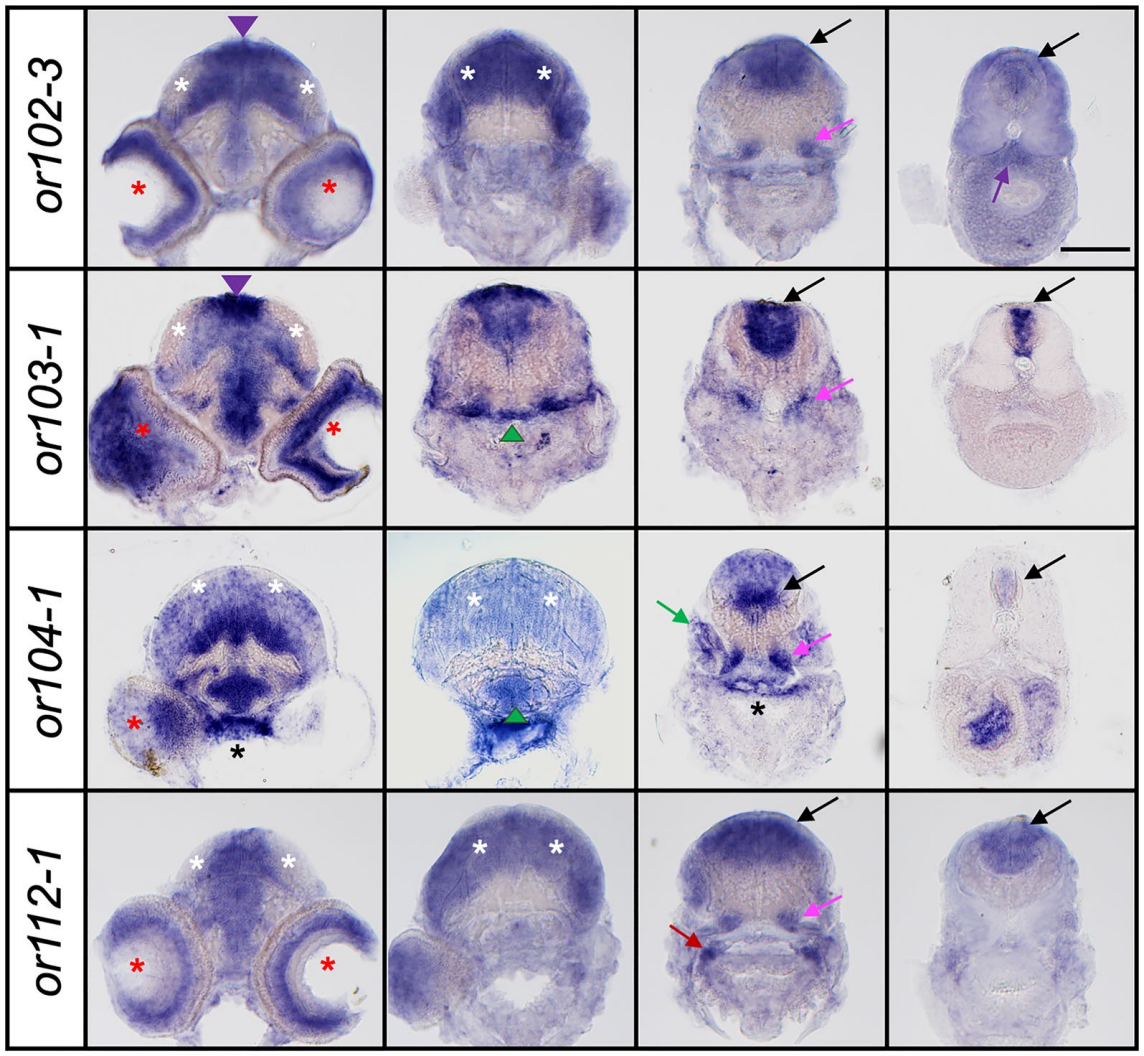
Spatial regulation of olfactory receptor gene expression during development. Representative images of transverse sections of 10-day-old larvae processed by in situ hybridization for *or102-3, or103-1, or104-1* and *or112-1.* Scale bar = 100 μm. Refer to Fig. 2 for symbol legends.

To test whether the expression pattern of OR genes was consistent during developmental stages, we also analyzed *or102-3*, *or103-1* and *or104-1* expression in 10-day-old larvae (Fig. 3). We found that the expression of each OR gene was similar to that observed in 6-day-old larvae. However, the difference in the intensity of the signals within the brain section was more pronounced at 10 days. For example, the labeling appeared stronger in the dorsal area of the mid- and hindbrain for *or103-1* while it was stronger in the ventral part of the hindbrain sections for *or104-1* (Fig. 3). We also analyzed the expression of a category 1 OR gene, *or112-1*, which in 6-day-old larvae shows little or no expression in the head. By contrast, we found that *or112-1* is highly expressed in the brain in 10-day-old larvae (Fig. 3). These results suggest that the OR genes are expressed differentially throughout development.

### RT-PCR amplification of olfactory receptor genes in non-olfactory tissues in larvae and adult zebrafish brain

**Figure 4:**
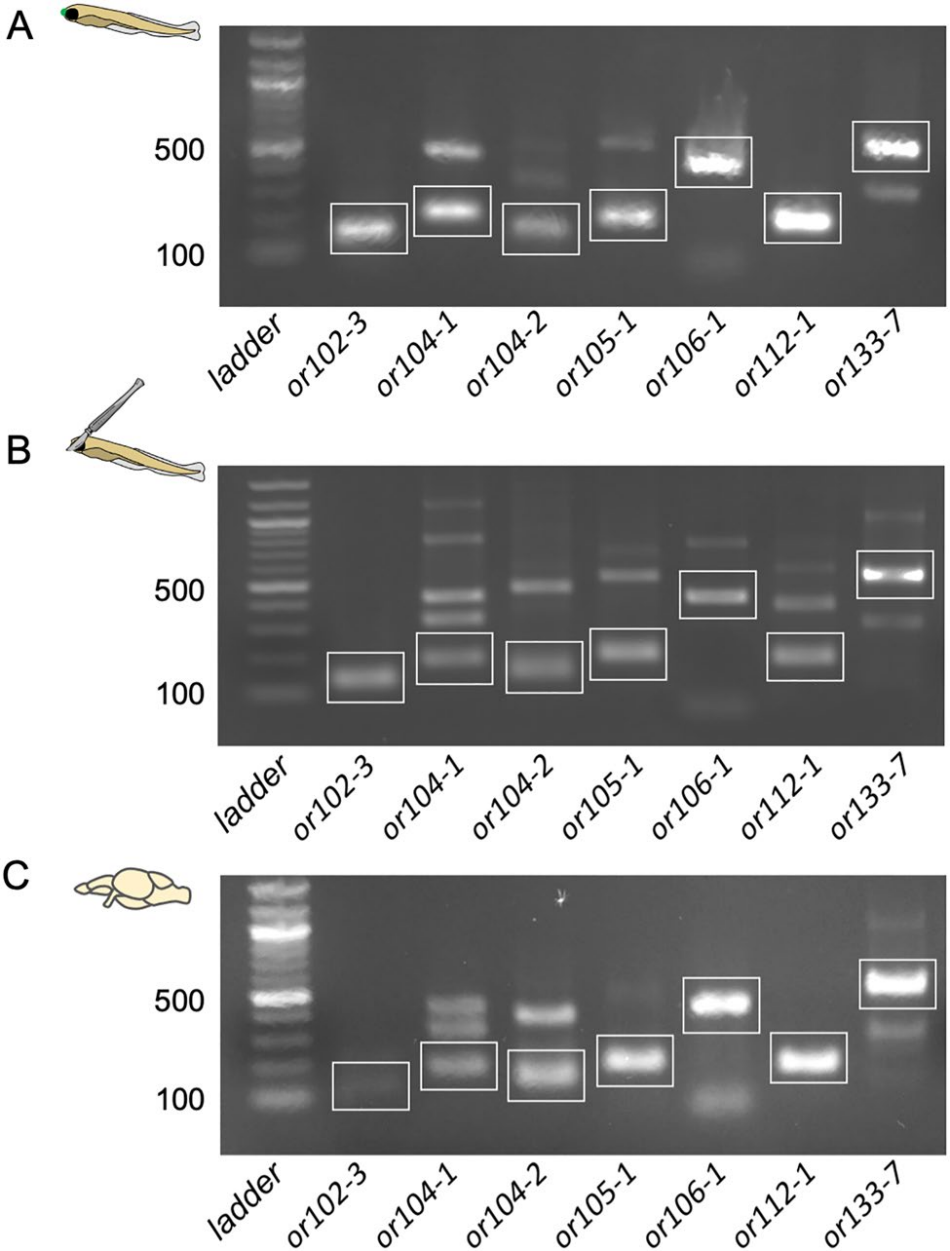
RT-PCR amplification of 7 olfactory receptor gene transcripts in non-olfactory tissues in larval and adult zebrafish. (**A**) *or102-3, or104-1, or104-2, or105-1, or106-1, or112-1* and *or133-7* genes are expressed in the whole 6-day-old zebrafish larvae. (**B**) All 7 OR gene transcripts are amplified in larvae without nose. 6-day-old *Tg(OMP: YFP)* larvae were used to ascertain the complete removal of the olfactory epithelium. (**C**) All tested OR gene transcripts are detected in the adult zebrafish brain. 100 bp ladder is used. Cartoons on left depict conditions for tissues used for RT-PCR. Green dot on rostral part of larva (**A**) represents OMP:YFP expressing olfactory epithelium, which was removed for (**B**). White boxes outline bands that correspond to the expected size. Original non-cropped images are in Fig. S3.

To confirm the expression of OR genes that was detected in the brain by ISH (*or102-3, or104-1, or104-2, or105-1, or106-1, or112-1 and or133-7*), we performed RT-PCR. As a positive control, we first amplified OR transcripts from cDNA extracted from whole larvae expressing yellow fluorescent protein (YFP) in the olfactory epithelium [*Tg(OMP:YFP*)^18^] (Fig. 4A). Next, we carried out RT-PCR from non-olfactory tissues from cDNA extracted from *Tg(OMP:YFP*)] larvae that had the fluorescent protein-expressing olfactory epithelium and its surrounding tissues removed. Indeed, we were also able to amplify all tested OR transcripts of expected sizes (Fig. 4B). Finally, we performed RT-PCR on cDNA from dissected adult zebrafish brains and were able to amplify the OR transcripts (Fig. 4C). The results confirm that OR genes are indeed expressed in non-olfactory tissues in the larvae and in the adult zebrafish brain.

### Olfactory receptor genes are expressed in specific structures in the adult brain

**Figure 5:**
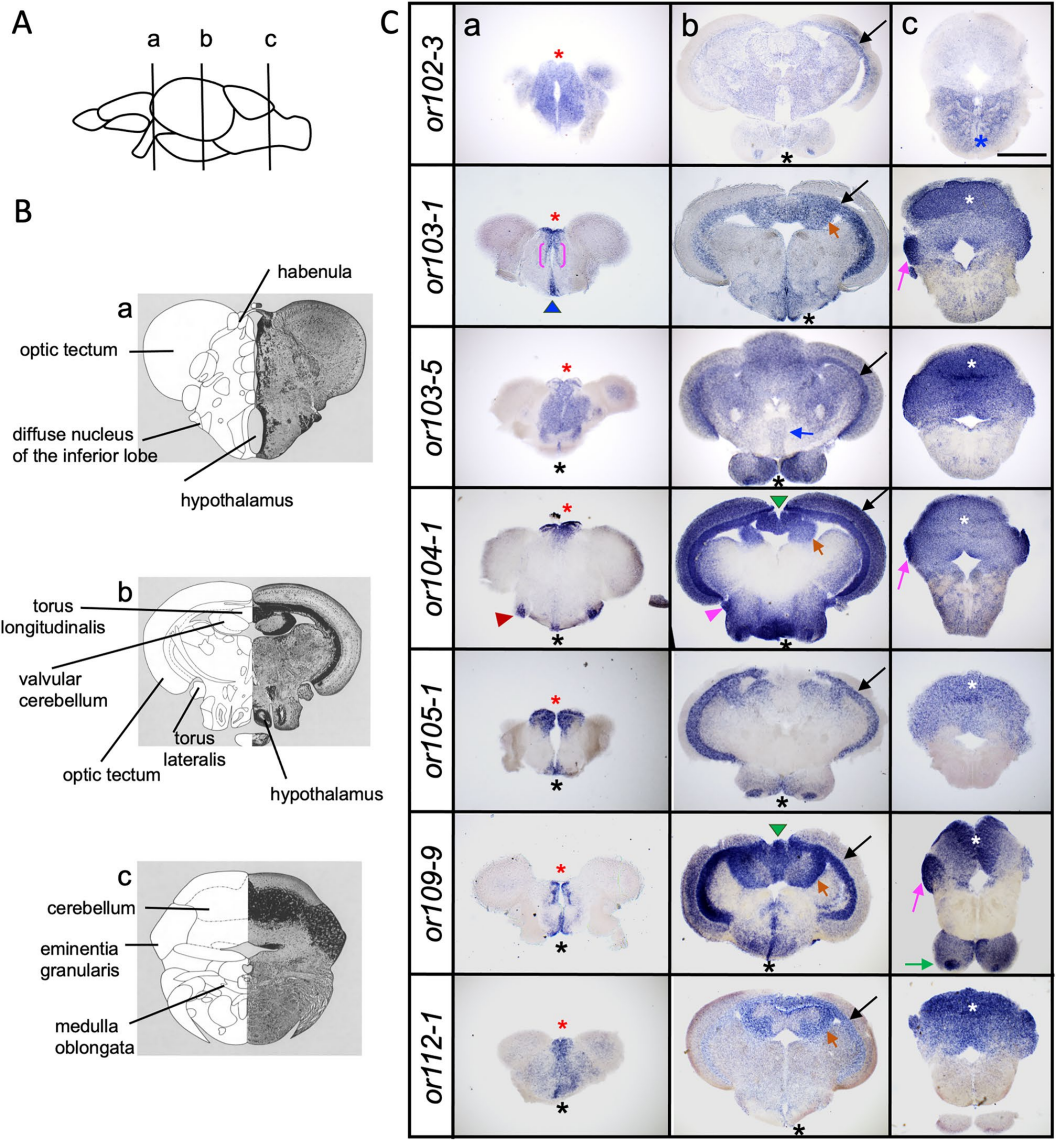
Olfactory receptor genes are expressed in the adult zebrafish brain. (**A**) Schematic drawing of a lateral view of the adult zebrafish brain indicating the anterior–posterior levels (a–c) of the transverse sections in (**B**) and (**C**). (**B**) Schematic image of transverse sections of adult zebrafish brain with different areas labeled in the forebrain (a), midbrain (b) and hindbrain (c) areas. Modified from Wulliman et al.^24^. (**C**) Representative transverse sections of adult zebrafish brains processed by in-situ hybridization for *or102-3*, *or103-1*, *or103-5*, *or104-1*, *or105-1*, *or109-9* and *or112-1* show different patterns of expression in distinct brain regions. Olfactory receptor gene transcripts are found in the habenula (red asterisk), preoptic area (blue arrowhead), hypothalamus (black asterisk), ventral thalamus (magenta bracket), diffuse nucleus of the inferior lobe (red arrowhead), optic tectum (black arrow), torus longitudinalis (green arrowhead), valvular cerebellum (orange arrow), eminentia granularis (magenta arrow), interpeduncular nucleus (blue arrow) cerebellum (white asterisk), medulla oblongata (blue asterisk), torus lateralis (magenta arrowhead). Scale bar = 500 μm.

Certain OR genes appeared to become more restricted in their expression profile during development (see Figs. 2 and 3). To test whether OR genes are expressed in specific identifiable brain regions in the adult, we carried out ISH for *or102-3, or103-1, or103-5, or104-1, or105-1, or109-9,* and *or112-1* in dissected adult brain sections. We analyzed their expression in the forebrain, midbrain and hindbrain regions. We found that all tested OR genes are expressed in the different brain regions, albeit with different patterns (Fig. 5). In the forebrain, we found that that all tested OR genes were expressed in the habenula (Fig. 5C, a). Interestingly, *or103-1, or103-5, 104-1, or105-1 and or109-9* were highly expressed in a restricted region in the habenula and the preoptic area (hypothalamus), while *or102-3* and *or112-1* were ubiquitously expressed throughout most of the forebrain*.* In the midbrain, all OR genes were expressed in the optic tectum as well as in the hypothalamus (Fig. 5C, b). Finally, in the hindbrain, *or102-3* was the only OR gene to be preferentially expressed in the ventral domain (medulla oblongata), while the rest of the OR genes were expressed largely in the dorsal domain (cerebellum). Interestingly, *or103-1* showed stronger staining in in the dorsal-most area of the hindbrain (Fig. 5C, c). Moreover, OR genes were also expressed in the diffuse nucleus of the inferior lobe (*or104-1*), eminentia granularis (*or103-1, or104-1, or109-9*), interpeduncular nucleus (*or103-5*), torus longitudinalis (*or104-1, or109-9*), valvular cerebellum (*or103-1, or104-1, or109-9, or112-1*) or the torus lateralis (*or103-1, or104-1*). These results indicate that specific structures in the adult brain express different combinations of OR genes.

## Discussion

### Conservation of olfactory receptor expression in non-olfactory tissues

The expression of ORs is not only restricted to the olfactory system, as many ORs are found widely spread throughout various tissues in rodent and humans^8^. However, the conservation of expression in other vertebrate animals nor the extent of the number of OR genes expressed in non-olfactory systems is unknown. To elucidate the spatial temporal conservation of OR gene expression in non-olfactory tissues, we carried out an ISH screen for 36 OR genes in larvae and 7 in the adult brain of zebrafish. We found that OR genes are indeed expressed in various tissues including the head, trunk and the gut areas. The OR genes that were expressed in the same areas were not part of the same OR subclass nor located on the same chromosome (data not shown). However, the exact number or subclass of OR genes that are expressed in non-olfactory tissues need to be further verified. Sequencing analysis revealed that the OR plasmids used to synthesize RNA probes were specific. However, it is difficult to ascertain the specificity of the anti-sense riboprobe binding to the endogenous mRNA, in particular to transcripts with highly similar sequences. ISH experiments using hybridization chain reaction (HCR)^25^ using multiple smaller probes to decrease background noise and increase specificity of the signal in future studies should resolve this issue.

Transverse sectioning of the head of larvae expressing 9 OR genes showed that they are found in various tissues including the eyes, roof of pharynx, brain and muscles. Sectioning adult brains also revealed that 7 OR genes are differentially expressed in distinct brain regions including the habenula, hypothalamus and the optic tectum. Further studies performing double-labeling using markers or transgenic lines labeling specific cellular populations, ganglions, muscles and pronephros will confirm the presence of the ORs in various tissues and brain nuclei.

### Dynamic expression of olfactory receptor genes in the brain

Expression of most ORs examined in the brain became more restricted during development. The expression of three OR genes that showed distinct patterns in the larval brain was assayed in 10-day-old larvae, in which neuronal reorganization is suspected to take place^26^. We found that while these OR genes were expressed in similar brain areas between the 6- and 10-day-old larvae, the spatial pattern became more restricted with age. By contrast, *or112-1* is weakly detected in 6-day-old larvae (Fig. 1, transverse sections not shown), while showing strong expression in the 10-day-old and adult brains. In the adult brain, the majority of the OR genes tested were expressed in the hypothalamus and habenula. 3 out of 5 hypothalamus-expressing OR transcripts are also observed in the hypothalamus in larvae. By contrast, none of the 7 adult habenula-expressing ORs were found in the larval habenula. These results suggest that the expression of ORs is highly regulated in a spatial and temporal manner.

In rodents, ORs are expressed in several brain areas, including the hypothalamus and the ventral tegmental area (VTA)^12^. In vitro studies showed response of OR-expressing dopaminergic neurons in the VTA to natural odorant molecules. However, the endogenous ligands that activate these receptors are unknown. It is noteworthy that all ORs assessed in the adult brain in this study show expression in the habenula and the hypothalamus. The hypothalamus has long been implicated in controlling food intake^27,28^ and recent studies have implicated the habenula in food-related behaviors^29–31^. One interesting possibility is that the odors during food intake is transported into the brain and plays a role in the modulation of brain areas associated with food-related behaviors.

### Implication for olfactory receptors in non-olfactory tissues

The endogenous ligands for the ORs are currently unknown^32^. Hence, it is difficult to hypothesize the role of these ORs in different tissues. However, the exposure to various environmental compounds that activate distinct ORs could result in the perturbation of cellular processes in cells that express specific OR genes. Indeed, in vitro studies on different cell types have shown that the activation of ORs affects distinct cellular processes. For example, Olfr78 found in murine kidney cells is activated by acetate and pronate and causes renin expression leading to vasoconstriction^33^. Moreover, Olfr2, which is expressed on macrophages, is activated by octanal resulting in increased inflammation and acceleration of atherosclerosis^34^. The upregulation of certain OR genes in colorectal or breast cancer cells is used as an early detection marker^35^. The activation of ORs in lung or prostate cancer cells results in apoptosis while in hepatocellular carcinoma, cell proliferation is reduced^13,14,36^. While certain chemicals have already been shown to induce specific cancer or health issues, ascertaining causality between environmental factors and most occupational diseases is often times contentious and inconclusive. Future studies identifying the external ligands for ORs expressed in specific cell types could provide a mechanistic understanding to how environmental factors affect human health.

### Breaking the code for ligand-receptor binding stereochemistry

GPCRs are the major targets for drug development due to their role in multiple diseases, however, deorphanizing (the process of identifying ligands) the GPCRs has not been straightforward^37^. This is more so an issue for the ORs, as the structure for vertebrate ORs has yet to be resolved. As one receptor can detect several odorant molecules and one odorant molecule can activate multiple ORs, the specificity of ligand-OR interaction is yet unclear^1^. As such, only 8–10% of mammalian ORs have been deorphanized^32^. In zebrafish, prostaglandinF2, the female sex pheromone has been shown to activate OR114-1^19^. Based on sequence homology, OR genes from terrestrial and aquatic animals have been categorized into distinct non-overlapping classes, in which the ligands were proposed to be air-borne volatile or water-soluble compounds, respectively^38^. However, terrestrial and aquatic animals show behavioral responses to the same molecules^39^, indicating that sequence homology in ORs does not determine ligand binding affinity. Prioritizing the identification of ORs that respond to molecules that elicit a behavioral response in both terrestrial and aquatic animals could provide the key to understanding the ligand-receptor stereochemistry using machine learning, which could be applied to design GPCR-targeted drugs^40,41^.

In conclusion, we show that multiple ORs are expressed throughout the body of zebrafish larvae and also in distinct areas in the adult brain. Despite the divergence in OR gene sequences between terrestrial and aquatic animals, the conservation of OR expression in non-olfactory tissues between vertebrate animals suggests that these ORs likely respond to similar odorants to mediate distinct cellular processes. As more species are constantly being subjected to unnatural environmental factors, it will be important to understand the role of ORs in non-olfactory tissues and how activation of these ORs could influence the health and well-being of the animal.

## Supplementary Information

Supplementary Information.

## Data Availability

The datasets used and/or analyzed during the current study are available from the corresponding author on request.
