# Supplementary material for: Expression of olfactory receptor genes in non-olfactory tissues in the developing and adult zebrafish
Source: Sci Rep. 2023 Mar 21;13:4651. doi: 10.1038/s41598-023-30895-3 (PMC10030859; doi:10.1038/s41598-023-30895-3)
Supplement: Supplementary file 1 — Supplementary Information. [file 41598_2023_30895_MOESM1_ESM.pdf]

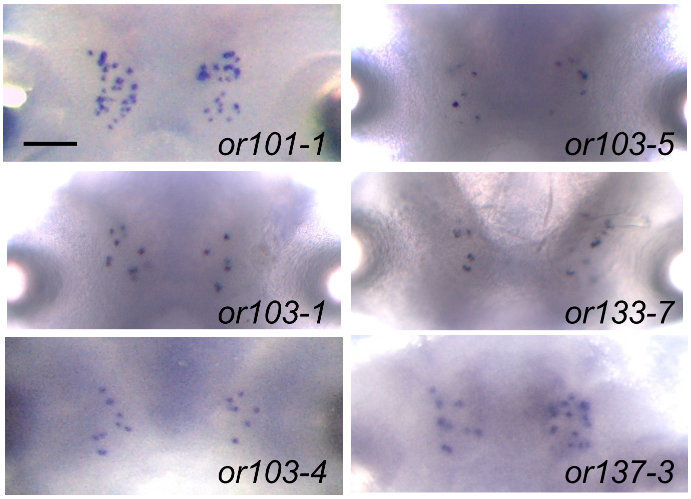

Figure S1. **Olfactory receptor genes are expressed in the olfactory epithelium.** Frontal views of 6-day-old larvae processed by whole mount in situ hybridization of selected olfactory receptor genes. Scale bar:100μm.

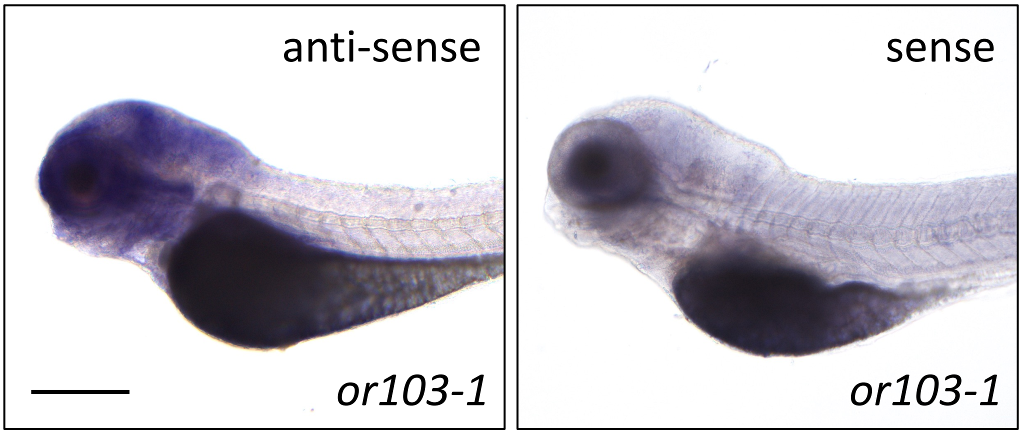

Figure S2. E**xpression of OR transcript in the brain is specific.** Lateral views of 6-day-old larvae processed by whole mount in situ hybridization using anti-sense (left) or sense (right) probes for *or103-1*. Scale bar: 200μm

	Olfactory epithelium	Head	Pharynx	Trunk	Pancreas
or101-1	■	■	■	■	
or102-3	■	■	■		■
or103-1	■	■	■	■	■
or103-5	■	■	■	■	■
or104-1	■	■	■		
or104-2	■	■	■	■	■
or105-1	■	■	■	■	
or106-1	■	■	■		■
or106-11	■	■			■
or108-1	■	■	■	■	■
or109-9	■	■		■	■
or111-1	■	■	■	■	■
or111-5	■	■		■	
or111-11	■				
or112-1	■				
or115-1	■				
or117-1	■	■	■		■
or120-1	■	■		■	
or125-4	■	■	■	■	■
or126-1	■	■		■	
or126-3	■				
or126-4	■	■		■	
or126-7	■	■		■	
or128-1	■	■	■	■	■
or128-4	■	■			
or128-5	■	■	■	■	■
or132-1	■	■	■	■	
or132-4	■		■		■
or133-2	■	■	■	■	
or133-4	■	■	■	■	■
or133-7	■	■		■	■
or133-10	■	■		■	■
or135-1	■	■	■	■	
or137-3	■	■	■	■	■
or103-4	■				
or137-9	■		■		■

Table S1. **Summary table of olfactory receptor gene expression in the zebrafish larvae.** Summary of 36 olfactory receptor gene expression pattern in different tissues (olfactory epithelium, head, pharynx, trunk and pancreas) in 6-day-old zebrafish larvae. See Figure 1.

A B

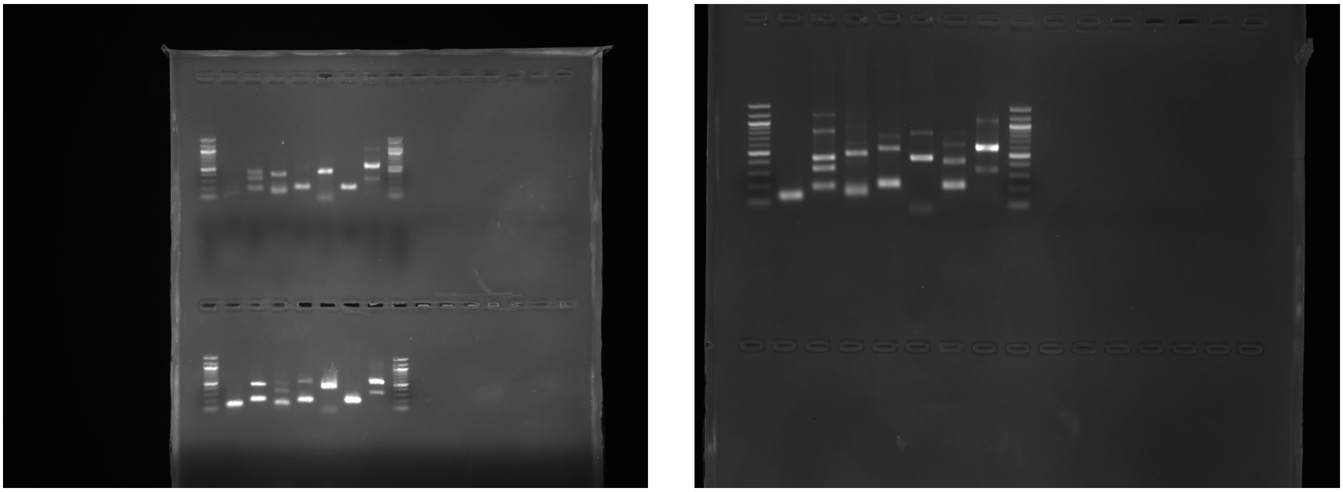

Figure S3. **Original images of the electrophoresis gel.** Bands from RT-PCR using cDNA from adult brains (A, top), whole larvae (A, bottom) or larvae with the OMP:GFP positive cells (olfactory epithelium) surgically removed (B) are shown.
